# Green Tea Extract Ameliorates Ischemia-Induced Retinal Ganglion Cell Degeneration in Rats

**DOI:** 10.1155/2019/8407206

**Published:** 2019-07-09

**Authors:** Yaping Yang, Ciyan Xu, Yuhong Chen, Jia-Jian Liang, Yanxuan Xu, Shao-Lang Chen, Shaofen Huang, Qichen Yang, Ling-Ping Cen, Chi Pui Pang, Xing-huai Sun, Tsz Kin Ng

**Affiliations:** ^1^Department of Ophthalmology and Vision Science, Eye and Ear Nose Throat Hospital, Shanghai Medical College, Fudan University, Shanghai, China; ^2^Joint Shantou International Eye Center of Shantou University and the Chinese University of Hong Kong, Shantou, Guangdong, China; ^3^Department of Ophthalmology & Visual Sciences, The Chinese University of Hong Kong, Hong Kong; ^4^Shantou University Medical College, Shantou, Guangdong, China

## Abstract

**Purpose:**

Oxidative stress induced by reduced blood circulation is a critical pathological damage to retinal ganglion cells (RGCs) in glaucoma. We previously showed that green tea extract (GTE) and its catechin constituents alleviate sodium iodate-induced retinal degeneration in rats. Here, we investigated the therapeutic effect of GTE on ischemia-induced RGC degeneration in rats.

**Methods:**

RGC degeneration was induced by ischemic reperfusion in adult Fischer F344 rats. Green tea extract (Theaphenon E) was intragastrically administered 4 times within 48 hours after ischemia. RGC survival, pupillary light reflex, expressions of cell apoptosis, oxidative stress, and inflammation-related proteins were studied.

**Results:**

Ischemic reperfusion significantly induced apoptotic RGCs, RGC loss, and larger constricted pupil area compared to the untreated normal rats. Expressions of activated caspase-3 and caspase-8, Sod2, and inflammation-related proteins as well as p38 phosphorylation were significantly upregulated in the ischemia-injured rats. Compared to the saline-fed ischemic rats, significantly higher number of surviving RGCs, less apoptotic RGCs, and smaller constricted pupil area were observed in the GTE-fed ischemic rats. GTE also reduced the increased protein expressions caused by ischemic injury but enhanced the Jak phosphorylation in the retina. Notably, green tea extract did not affect the survival of RGCs in the uninjured normal rats.

**Conclusions:**

In summary, GTE offers neuroprotection to RGCs under ischemic challenge, suggesting a potential therapeutic strategy for glaucoma and optic neuropathies.

## 1. Introduction

Glaucoma is a leading cause of irreversible blindness and visual impairment in most populations, affecting 80 million people worldwide [1]. It is characterized by progressive retinal ganglion cell (RGC) and axonal degeneration. While intraocular pressure (IOP) elevation has been proven to be associated with RGC degeneration, retinal ischemic insults, commonly occurred in acute angle closure and retinal vascular occlusion, can also contribute to the loss of RGCs. Optical coherence tomography (OCT) angiography revealed lower vessel density in primary angle closure glaucoma patients [2]. The reduced blood circulation is predisposition to oxidative stress, which is a critical initiation for RGC degeneration [3]. Oxidative stress induced by reactive oxygen species (ROS) can initiate the caspase-mediated apoptotic pathway through lipid peroxidation, disruption of mitochondrial functions, and DNA damage [4]. Moreover, ROS and the oxidative processes can stimulate the generation of advanced glycation end products, which induce the release of proinflammatory cytokines, such as tumor necrosis factor- (TNF-) *α* and interleukin- (IL-) 6 [5]. In the ischemic reperfusion model, we have identified that the involvement of inflammatory response and the JAK/STAT pathway are important for RGC survival regulation [6, 7].

Green tea extract (GTE) possesses antioxidative, anti-inflammatory, and antiangiogenic properties [8, 9]. Its catechin constituent, (-)-epigallocatechin gallate (EGCG), could reduce retinal damages induced by ischemia/reperfusion [10]. EGCG can also protect photoreceptor cells from light- and sodium nitroprusside-induced degeneration in the rodent models [11, 12]. We reported that sodium iodate induces RPE damage and photoreceptor disruption by oxidative stress [13] and green tea extract and EGCG can attenuate sodium iodate-induced retinal degeneration in rats [14]. We had found that GTE shows better neuroprotective effect than EGCG [14]. The effect of GTE on RGC degeneration is unknown. Herein, we hypothesized that green tea extract could ameliorate ischemia-induced RGC degeneration *in vivo*. In this study, RGC degeneration in rats was induced by ischemic reperfusion, and the ischemia-injured rats were intragastrically fed with GTE. The RGC survival and the pupillary light reflex were determined. The expressions of cell apoptosis, oxidative stress, and inflammation-related proteins were evaluated.

## 2. Material and Methods

### 2.1. Animals

Female Fischer F344 rats (6–8 weeks old, weighing 200–250 g), which can be better anesthetized under the 2-hour ischemia injury period than the male rats, were purchased from Beijing Vital River Laboratory Animal Technology Co. Ltd., China. The rats were maintained at 22 ± 1°C, 40 ± 10% humidity, and with 12 : 12 hour dark-light cycle. Standard rodent chow and water were provided *ad libitum*. All rats were treated according to the Guidelines of the Association for Research in Vision and Ophthalmology (ARVO) Statement on the Use of Animals in Ophthalmic and Vision Research. The experimental protocols in this study have been approved by the Animal Experimentation Ethics Committees of Fudan University, the Joint Shantou International Eye Center of Shantou University, and the Chinese University of Hong Kong. For each experimental group, 5 rats were used. There is no gender difference in the ischemia-induced RGC degeneration model.

### 2.2. Ischemic Reperfusion

Ischemic reperfusion was performed in rats according to our previous studies [6, 7]. Briefly, the rats were anesthetized by intraperitoneal injection of 1 : 1 mixture (1.5 ml/kg) of ketamine (100 mg/ml) and xylazine (20 mg/ml). A 27-gauge needle was inserted into the anterior chamber of the left eye, and the needle was connected to a container carrying 500 ml sterile saline. The container was raised to a height of 1.5 m above the eye to elevate the IOP to 110 mmHg. Ischemic reperfusion was maintained for 2 hours. The IOP was monitored by Tonolab (Icare®, Finland) to ensure sustained induction of ischemic reperfusion. The control group was the rats without ischemic reperfusion surgery.

### 2.3. Green Tea Extract Treatment

**Figure 1:**
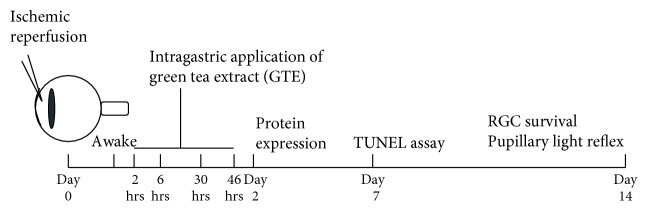
Schematic diagram of ischemic reperfusion, green tea extract treatments, and experimental assessments in rats. The rats were anesthetized, and ischemic reperfusion injury was induced by the infusion of sterile saline for 2 hours with IOP elevation to 75 mmHg. The rats were intragastrically fed 4 times with 275 mg/kg green tea extract (GTE) at 2, 6, 30, and 46 hours after ischemic reperfusion injury. Protein expression was evaluated on day 2. Retinal ganglion cell (RGC) survival and function were determined on day 14 after ischemic reperfusion injury.

Green tea extract (Theaphenon E) was generously provided by Dr. Yukihiko Hara (Department of Environmental Physiology, Shimane University Faculty of Medicine, Japan). We have previously characterized the proportion of each catechin constituent in Theaphenon E (EGCG: 70.53%, EGC: 4.61%, EC: 3.88%, and GC: 0.64%) [15]. Green tea extract was suspended in distilled water freshly before application. The rats were intragastrically fed 4 times with 275 mg/kg green tea extract at 2, 6, 30, and 46 hours after ischemic reperfusion (Figure 1). The dosage of GTE was selected according to our previous studies [8, 14]. Rats in the control group were fed with the same volume of normal saline. The rats were maintained for 2 weeks before further analyses.

### 2.4. Retinal Ganglion Cell Survival Analysis

The survival of RGC was evaluated by immunofluorescence according to our established protocol [16]. On postsurgery day 14, which the number of surviving RGC is stable, the rats were terminated with overdose of sodium pentobarbital (20%, *w*/*v*) and perfused with phosphate-buffered saline (PBS) followed by 4% paraformaldehyde (Sigma-Aldrich) in PBS at pH 7.4. The eyes were removed and further fixed in 4% paraformaldehyde overnight. After postfixation, the retinas were dissected out from the eyeballs, followed by three times of 5-minute PBS washing. After being blocked and permeabilized with 10% normal goat serum (Biodesign, Saco, ME) and 0.2% Triton X-100 for 1 hour, the retinas were incubated overnight at 4°C with adult RGC-specific anti-neuron-specific *β*III-tubulin antibody (1 : 500; COVANCE, Emeryville, CA), which can label and quantitatively represent the surviving RGC [16]. The retinas were then washed with PBS (5 minutes for 3 times) and incubated with Cy3-conjugated secondary antibody (1 : 400; Jackson Laboratory, Bar Harbor, ME) overnight at 4°C. After 3 times of 5-minute PBS wash, the retinas were mounted with antifading mounting medium (Dako Corporation, Carpinteria, CA) and imaged under a confocal microscope (Leica TCS SP5 II, Leica Microsystems, Wetzlar, Germany). To determine the number of positive cells in each retina, the number of positively stained cells was counted in 30 fields (0.25 × 0.25 mm^2^ each) per retina at a fixed distance in a pattern of grid intersections. The average density of positive cells was determined.

### 2.5. Retinal Ganglion Cell Function Analysis

The RGC function of the rats could be evaluated by their pupillary light reflex measured by pupil constriction [17]. Briefly, the rats were dark-adapted for 30 min and maintained under anesthesia during examination. The stimulus was provided by a halogen light source (100 W, 12 V) from a surgical microscope (Topcon, Japan). The light source was maintained to focus with 8x objective and aligned perpendicular to the center of the pupil. The stimulus time was 10 sec, followed by 60 sec of darkness to restore the pupil diameter to baseline level. The pupil status was recorded with the WinFast PVR photography system. The constricted pupil diameter and area were measured by ImageJ (version 1.47; NIH, Bethesda, MD). The pupil diameter of rat under bright light is around 0.2 mm.

### 2.6. Apoptosis Analysis

RGC apoptosis was evaluated by the TUNEL assay using the DeadEnd™ colorimetric TUNEL system (Promega, Madison, MI). On postsurgery day 7, which the RGC death starts, the rats were terminated with overdose of sodium pentobarbital and perfused with PBS followed by 4% paraformaldehyde in PBS at pH 7.4. The eyes were removed and further fixed in 4% paraformaldehyde overnight. After postfixation, the eyeballs were cryoprotected with 10-30% sucrose gradient in PBS. The eyeball slices (10 *μ*m) were sectioned using the vibratome (Leica), permeabilized with 0.2% Triton® X-100 solution (Sigma-Aldrich) in PBS for 5 minutes at room temperature, and incubated in a TUNEL reaction mixture at 37°C for 1 hour. The TUNEL-labeled cells were imaged on a confocal microscope (Leica TCS SP5 II) and analyzed by the ImageJ software. For each sample, 5 retinal sections were used for the TUNEL analysis and 10 fields were imaged.

### 2.7. Immunoblotting Analysis

On posttreatment day 2, which the molecular signals are critical for RGC survival, the retina was lysed with the RIPA buffer (Sigma-Aldrich) supplemented with protease and phosphatase inhibitors (Roche). Total protein concentrations of the cell lysates were measured by protein assay (Bio-Rad). After denaturation, equal amount of total proteins (20 *μ*g) was resolved in 12.5% SDS-polyacrylamide gel and electrotransferred to nitrocellulose membranes for sequential probing with the primary antibodies for cell apoptosis (cleaved caspase-3 (Casp3) and caspase-8 (Casp8)), survival signal (total and phospho-Jak), oxidative stress (total and phospho-p38 and superoxide dismutase 2 (Sod2), total), and inflammation (toll-like receptor 4 (TLR4), Il-1*β* and TNF-*α*) and respective secondary antibodies conjugated with horseradish peroxidase (Santa Cruz Biotechnology). Signals were detected by enhanced chemiluminescence (Amersham Pharmacia, Cleveland, OH) with the ChemiDoc™ XRS+ system (Bio-Rad). *β*-Actin was the housekeeping protein.

### 2.8. Statistical Analysis

The data was presented as the mean of the results from 5 rats ± standard deviation (SD). One-way analysis of variance (ANOVA) with post hoc Tukey test (for multiple testing correction) was used to compare the means among different treatment groups. All statistical analyses were performed by the commercially available software (IBM SPSS Statistics 21; SPSS Inc., Chicago, IL). Significance was defined as *p* < 0.05.

## 3. Results

### 3.1. Green Tea Extract Ameliorated Ischemia-Induced Retinal Ganglion Cell Degeneration

**Figure 2:**
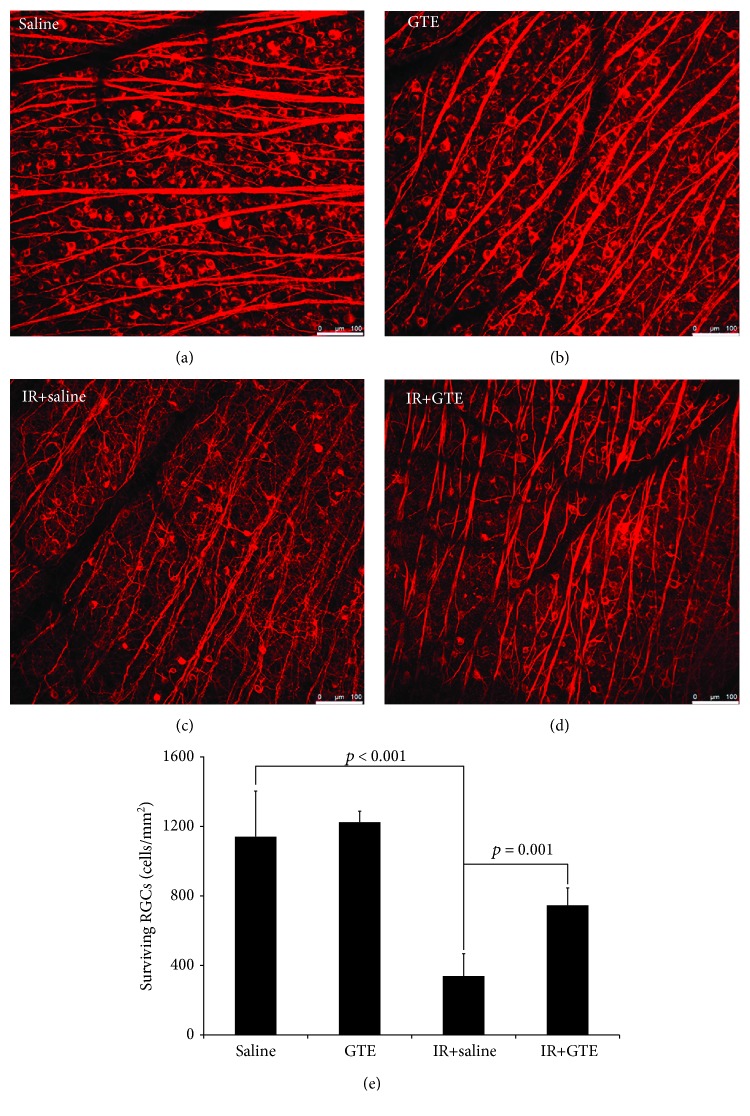
Retinal ganglion cell survival analysis for the green tea extract treatment effect on normal and ischemia-injured rats. Retinal ganglion cell (RGC) survival was evaluated by the immunofluorescence analysis of retinal wholemount for *β*III-tubulin on day 14 after ischemic reperfusion (IR) injury. (a) Surviving RGCs in the saline-fed normal rats. (b) Surviving RGCs in the green tea extract- (GTE-) fed normal rats. (c) Surviving RGCs in the saline-fed rats with ischemic injury. (d) Surviving RGCs in the GTE-fed rats with ischemic injury. (e) Quantitative analysis of surviving RGCs in the retina. Data was presented as mean ± standard deviation. Scale bar: 100 *μ*m.

Four-time intragastric administrations of GTE in a 2-day treatment period (Figure 1) did not influence RGC survival in normal rats (Figures 2(a) and 2(b)). The number of surviving RGCs in the GTE-fed normal rats at 2 weeks after GTE ingestion (1224.6 ± 63.6 cells/mm^2^) was not statistically different from that of the saline-fed normal rats (1141.7 ± 262.0 cells/mm^2^, *p* = 0.771; Figure 2(e)). Systemic influence or severe adverse event was not observed from the GTE-fed normal rats along the 2-week observation period. Our results indicated that oral administration of 275 mg/kg GTE is not toxic to the retina, indicating that this dosage of GTE could be used for further study.

Ischemic reperfusion, with an acute IOP elevation of 110 mmHg for 2 hours, dramatically reduced the survival of RGCs (Figure 2(c)). Compared to the uninjured normal rats, the number of surviving RGCs in the retina of the ischemia-injured rats was significantly decreased (339.1 ± 128.7 cells/mm^2^, *p* < 0.001; Figure 2(e)). Intragastric ingestion of GTE effectively ameliorated the ischemia-induced RGC degeneration (Figure 2(d)). Significantly higher number of surviving RGCs was found in the retina of the ischemia-injured rats fed with GTE (746.0 ± 100.2 cells/mm^2^, *p* = 0.001) when compared to that fed with saline (Figure 2(e)). Therefore, our results indicated that GTE could promote survival of RGCs after ischemia injury.

### 3.2. Green Tea Extract Suppressed the Pupillary Light Reflex Impairment after Ischemia Injury

**Figure 3:**
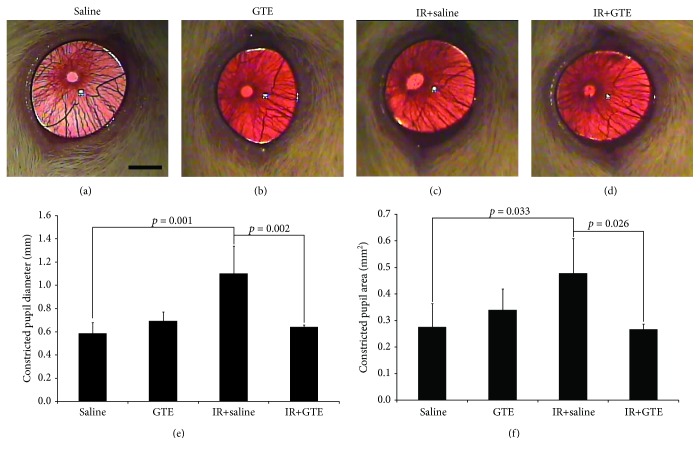
Pupillary light reflex analysis for the green tea extract treatment effect on normal and ischemia-injured rats. Retinal ganglion cell (RGC) function was evaluated by the pupillary light reflex on day 14 after ischemic reperfusion (IR) injury. Pupillary light reflex was measured by the pupil constriction. (a) Surviving RGCs in the saline-fed normal rats. (b) Surviving RGCs in the green tea extract- (GTE-) fed normal rats. (c) Surviving RGCs in the saline-fed rats with ischemic injury. (d) Surviving RGCs in the GTE-fed rats with ischemic injury. (e) Quantitative analysis of the constricted pupil diameter. (f) Quantitative analysis of the constricted pupil area. Data was presented as mean ± standard deviation. Scale bar: 2 mm.

To confirm the rescue effect of GTE on ischemia-induced RGC degeneration, we evaluated the pupillary light reflex by measuring the pupil constriction in rats after ischemia injury. GTE treatment in normal rats did not affect the reflex action in response to light (Figures 3(a) and 3(b)). The constricted pupil diameter (0.69 ± 0.08 mm) and the constricted pupil area (0.34 ± 0.08 mm^2^) of the GTE-fed normal rats at 2 weeks after GTE treatment did not show a statistically significant difference from that of the saline-fed normal rats (diameter: 0.59 ± 0.09 mm, *p* = 0.703; area: 0.28 ± 0.09 mm^2^, *p* = 0.751; Figures 3(e) and 3(f)). The reduced RGC survival after ischemic reperfusion could be correlated with the weakened reflex action in response to light (Figure 3(c)). Compared to the uninjured normal rats, significantly longer constricted pupil diameter (1.10 ± 0.24 mm, *p* = 0.001) and larger constricted pupil area (0.48 ± 0.13 mm^2^, *p* = 0.033) were observed from the ischemia-injured rats. On the contrary, GTE treatment after ischemia injury effectively suppressed the reflex action impairment in response to light (Figure 3(d)). The constricted pupil diameter (0.64 ± 0.02 mm, *p* = 0.002) and the constricted pupil area (0.27 ± 0.02 mm^2^, *p* = 0.026) were significantly reduced in the ischemia-injured rats fed with GTE when compared to the saline-fed ischemia-injured rats. Collectively, our results indicated that ischemia-induced RGC degeneration abolished the RGC function and GTE suppressed the RGC function impairment after ischemia injury.

### 3.3. Green Tea Extract Repressed Ischemia-Induced Apoptosis, Oxidative Stress, and Inflammation in Retinal Ganglion Cells

**Figure 4:**
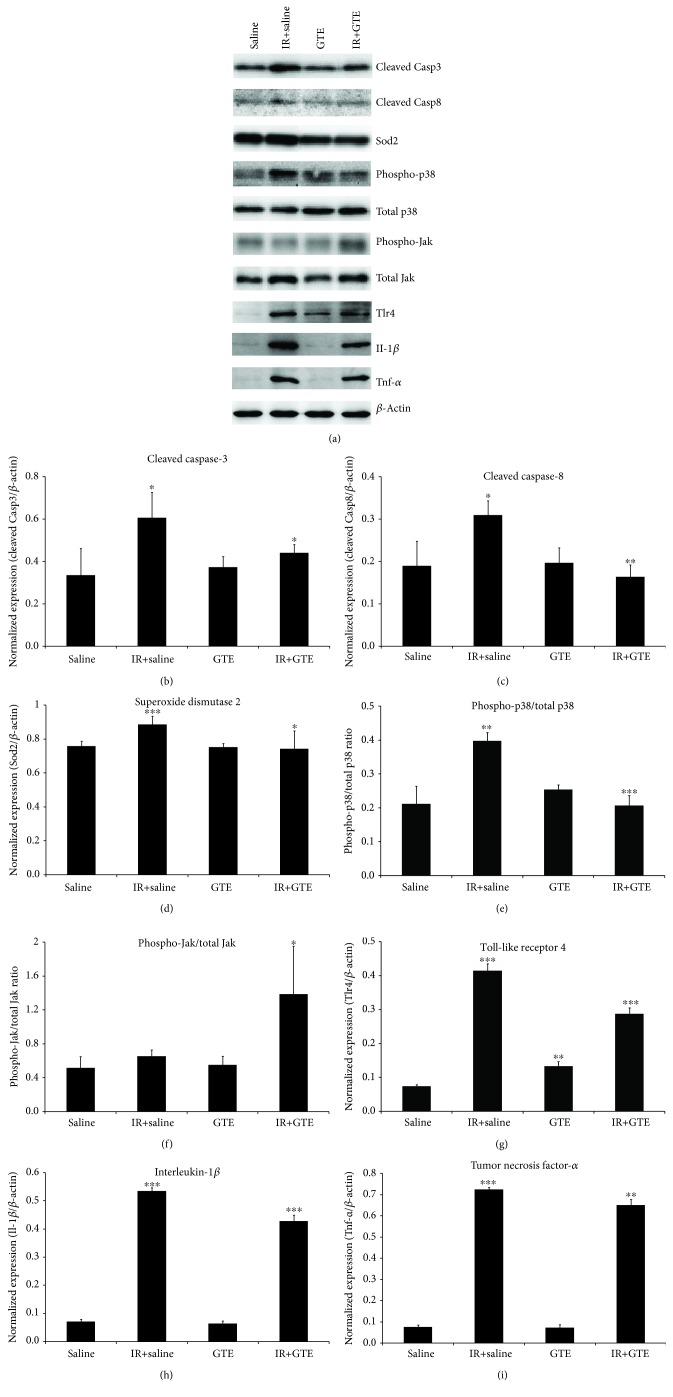
The expression analysis of cell apoptosis, oxidative stress, and inflammation-related proteins in the retina of ischemia-injured rats with green tea extract treatment. (a) Protein expression was evaluated by the immunoblotting analysis on day 2 after ischemic reperfusion injury. Relative fold change analyses on (b) caspase-8 (Casp8), (c) toll-like receptor 4 (TLR4), (d) interleukin-1*β* (Il-1*β*), (e) tumor necrosis factor-*α* (TNF-*α*), (f) phospho-p38 (p-p38), and (g) phospho-Jak (p-Jak). *β*-Actin was used as housekeeping protein for normalization. Data was presented as mean ± standard deviation. Compared to the saline-fed normal rats: ^∗∗^*p* < 0.01, ^∗∗∗^*p* < 0.001. Compared to the saline-fed ischemia-injured rats: ^#^*p* < 0.05, ^##^*p* < 0.01, ^###^*p* < 0.001.

To determine the mechanism of the GTE treatment effect on ischemia-induced RGC degeneration, we examined the protein expression of apoptosis, oxidative stress, and inflammation-related markers by the immunoblotting analysis (Figure 4(a)). The increased expression of apoptosis-related proteins cleaved Casp3 and Casp8 was found in the retina of the ischemia-injured rats (1.81 and 1.63 folds, respectively, *p* < 0.05) when compared to that of the uninjured normal rats (Figures 4(b) and 4(c)). In contrast, compared to the saline-fed rats with ischemic reperfusion, the expressions of cleaved Casp3 and Casp8 were reduced in the GTE-fed rats after ischemic injury by 1.37 and 1.89 folds (*p* < 0.05), respectively. Similarly, the expressions of inflammation-related proteins (TLR4, Il-1*β*, and TNF-*α*) were significantly upregulated in the retina of the ischemia-injured rats by 5.61, 7.46, and 9.62 folds (*p* < 0.001), respectively, compared to the uninjured normal rats (Figures 4(g)–4(i)). GTE treatment significantly downregulated the expressions of TLR4, Il-1*β*, and TNF-*α* proteins in the retina by 1.44, 1.25, and 1.11 folds (*p* < 0.01), respectively, compared to the saline-fed rats after ischemic injury. Besides, the expression of oxidative stress-related signaling protein Sod2 and the phospho-p38/total p38 ratio was also significantly upregulated in the retina of the ischemia-injured rats by 1.17 and 1.88 folds (*p* < 0.01), respectively, compared to the uninjured normal rats (Figures 4(d) and 4(e)). GTE treatment significantly downregulated the expressions of Sod2 and the phospho-p38/total p38 ratio in the retina by 1.19 and 1.93 folds (*p* < 0.05), respectively. In contrast, the survival signal phospho-Jak/total Jak ratio was significantly upregulated in the retina of the GTE-fed rats by 2.12 folds (*p* < 0.05), compared to the saline-fed rats. Expressions of cleaved Casp3, Casp8, Il-1*β*, and TNF-*α* as well as the ratios of phospho-p38/total p38 and phospho-Jak/total Jak in the retina of the GTE-fed normal rats were not significantly changed, compared to that of the saline-fed normal rats (*p* > 0.05; Figures 4(a)–4(i)).

**Figure 5:**
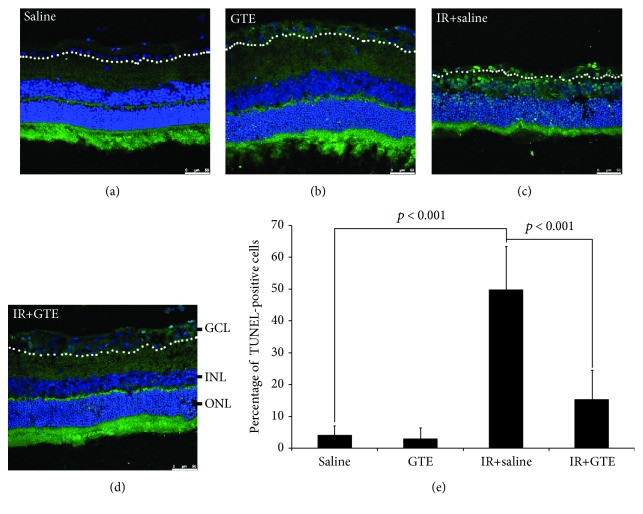
Apoptosis analysis of retinal ganglion cells after the green tea extract treatment effect on normal and ischemia-injured rats. Retinal ganglion cell (RGC) apoptosis was evaluated by the TUNEL analysis on retinal sections on day 7 after ischemic reperfusion (IR) injury in (a) the saline-fed normal rats, (b) green tea extract- (GTE-) fed normal rats, (c) saline-fed rats with ischemic injury, and (d) GTE-fed rats with ischemic injury. ONL: outer nuclear layer; INL: inner nuclear layer; GCL: ganglion cell layer (on the white-dotted line); green: TUNEL (apoptotic cells); blue: DAPI (nucleus). (e) Quantitative analysis of TUNEL-positive cells in the GCL. Data was presented as mean ± standard deviation. Scale bar: 50 *μ*m.

To confirm the antiapoptotic effect of GTE, we evaluated the apoptosis rate in the retina after ischemia injury and GTE treatment. The TUNEL assay showed that the number of TUNEL-positive cells in the ganglion cell layer (GCL) of the ischemia-injured rats (49.88 ± 13.61%; Figure 5(c)) was significantly higher than that in the uninjured normal rats (4.15 ± 2.90%, *p* < 0.001; Figures 5(a) and 5(e)). On the contrary, GTE treatment significantly reduced the number of TUNEL-positive cells in the GCL after ischemia injury (15.40 ± 9.12%, *p* < 0.001; Figures 5(d) and 5(e)), compared to that fed with saline. Collectively, our results indicated that ischemic reperfusion induced RGC degeneration through induction of cell apoptosis, oxidative stress, and inflammation, and the promotion of RGC survival by GTE after ischemia injury could be mediated by reducing cell apoptosis, oxidative stress, and inflammation as well as enhancing the survival signal.

## 4. Discussion

In this current study, our results demonstrated that (1) oral administration of 275 mg/kg GTE (Theaphenon E) is safe and would not induce toxic effect to the retina in rats; (2) ischemic reperfusion induces RGC degeneration in rats; (3) ischemia injury induces cell apoptosis, oxidative stress, and inflammation in the retina; (4) GTE treatment effectively alleviates ischemia-induced RGC degeneration; (5) GTE treatment suppresses the pupillary light reflex and RGC function impairment in rats after ischemic injury; and (6) green tea extract treatment reduces cell apoptosis, oxidative stress, and inflammation and increases survival signal in the retina. Collectively, these findings reveal a potent protective effect of GTE on RGC degeneration caused by ischemia and suggest a potential therapeutic application of GTE for glaucoma.

Ischemic injury is known to induce oxidative stress in the retina and consequently RGC degeneration [18]. In this study, we showed that Sod2 expression and p38 phosphorylation were upregulated in the retina after ischemic reperfusion, whereas GTE could reduce the increased Sod2 expression and p38 phosphorylation after ischemic reperfusion (Figures 4(d) and 4(e)). Since Sod is an oxidative stress marker [14] and oxidative stress alleviation is associated with p38 phosphorylation suppression [19], ischemic reperfusion induces oxidative stress in the retina, and GTE could relieve the oxidative stress induced by ischemic reperfusion. Similarly, our previous study also demonstrated that GTE can ameliorate sodium iodate-induced retinal degeneration by reducing the oxidative stress [14]. Because of the oxidative stress induction, multiple antioxidation strategies have been tested in the ischemic reperfusion model. Coenzyme Q10, carotenoid derivative, and flavonoid have been shown to prevent RGC loss in rodents by reducing the oxidative stress [20–22]. Moreover, green tea catechin EGCG has also been reported to revert the RGC layer thinning, reduced the *Thy1* gene expression, and increased the *Casp8* gene expression in rats with ischemic reperfusion [10]. In this study, we, for the first time, demonstrated that postsurgical intragastric administration of GTE promoted the survival of RGCs against degeneration induced by ischemic reperfusion (Figure 2). Besides, GTE suppressed the pupillary light reflex impairment (Figure 3), which confirms the neuroprotective effect of GTE. Furthermore, we observed that GTE reduced the ischemia-induced increase in apoptosis, including the cleaved Casp3 and Casp8 protein expression (Figures 4(b) and 4(c)) as well as the TUNEL-positive cells in the GCL (Figure 5). Coherently, inhibition of casepase-8 activation has also been reported in ischemia-injured mice treated with kaempferol [23]. In addition, we observed the increased Jak activation in the retina of the GTE-fed rats (Figure 4(f)). As Jak mediates the protective effect of RGCs [24], our findings suggest that GTE can enhance the survival signal in the retina to promote RGC survival. These indicate that ischemic reperfusion induces RGC degeneration through cell apoptosis and green tea extract could reduce cell apoptosis and enhance the survival signal in ischemic retina. Collectively, the enhanced RGC survival by GTE should be attributed to reductions in cell apoptosis and oxidative stress and the increase in survival signal.

Apart from oxidative stress and cell apoptosis, inflammation is also involved in ischemic reperfusion. Pretreatment of dexmedetomidine reduced retinal ischemic reperfusion injury by inhibiting inflammatory response through the TLR4 pathway [25], whereas phosphodiesterase III inhibitor prevented retinal ischemic damage through the inhibition of TNF-*α* [26]. In this study, we confirmed the induction of inflammatory response after ischemic reperfusion. The expressions of TLR4, Il-1*β*, and TNF-*α* proteins were upregulated in the retina after ischemic reperfusion injury (Figures 4(g)–4(i)). GTE treatment reduced the ischemia-induced inflammatory response in that the increased expressions of TLR4, Il-1*β*, and TNF-*α* proteins after ischemic reperfusion which were reduced in the GTE-fed rats. This is consistent to the results of our previous study which showed that GTE attenuated the inflammatory response induced by lipopolysacchride in the rat retina [27]. Therefore, the reduced inflammatory response by GTE could contribute to the enhanced RGC survival after ischemic reperfusion injury. Notably, in the GTE-fed uninjured rats, we observed an upregulation of the TLR4 expression in the retina (Figure 4(g)). Although EGCG has been shown to suppress the TLR4 expression through the 67 kDa laminin receptor [28], other components in GTE might have the potential to upregulate the TLR4 expression in the retina. Further investigations should delineate how different components in GTE interact with TLR4 in the retina and the target cells in the inflammatory response regulation by GTE.

In summary, the results from our *in vivo* investigations revealed that oral intake of GTE is safe and can ameliorate ischemia-induced RGC degeneration and suppress RGC function impairment in rats through the alleviation of cell apoptosis, oxidative stress, and inflammation. Since GTE shows a better protective effect than EGCG in our previous retinal degeneration model [14] and green tea catechins can be found within hours after GTE oral administration [15], this study further supports that GTE can serve as a potent therapeutic agent to RGC degeneration and support the report that tea consumption could possibly be protective against glaucoma development [29].

## Data Availability

The data used to support the findings of this study are available from the corresponding authors upon request.
